# A high M1/M2 ratio of tumor-associated macrophages is associated with extended survival in ovarian cancer patients

**DOI:** 10.1186/1757-2215-7-19

**Published:** 2014-02-08

**Authors:** Meiying Zhang, Yifeng He, Xiangjun Sun, Qing Li, Wenjing Wang, Aimin Zhao, Wen Di

**Affiliations:** 1 Department of Obstetrics and Gynecology, Ren Ji Hospital, School of Medicine, Shanghai Jiao Tong University, 160 Pujian Road, Shanghai 200127, China; 2 Shanghai Key Laboratory of Gynecologic Oncology, Ren Ji Hospital, School of Medicine, Shanghai Jiao Tong University, 160 Pujian Road, Shanghai 200127, China

**Keywords:** Ovarian cancer, Tumor-associated macrophage, M1, M2, Prognosis

## Abstract

**Background:**

Tumor-associated macrophages (TAMs) are classified into two major phenotypes, M1 and M2. M1 TAMs suppress cancer progression, while M2 TAMs promote it. However, little is known regarding the role of TAMs in the development of ovarian cancer. Here, we investigated the relationship between TAM distribution patterns (density, microlocalization, and differentiation) and ovarian cancer histotypes, and we explored whether altered TAM distribution patterns influence long-term outcomes in ovarian cancer patients.

**Methods:**

A total of 112 ovarian cancer patients were enrolled in this study, and the subjects were divided into two groups according to their survival (< 5 years vs. ≥ 5 years). Immunohistochemistry and immunofluorescence were used to determine the density, microlocalization, and differentiation status of TAMs in ovarian cancer tissues for each histotype. Kaplan-Meier survival and multivariate Cox regression analyses were used to evaluate the prognostic significance of TAM-related parameters in ovarian cancer.

**Results:**

TAMs most frequently infiltrated into the cancer tissue of the serous histotype, followed by mucinous, undifferentiated, endometrioid, and clear cell histotypes (p = 0.049). The islet/stroma ratio of total TAMs varied among the cancer histotypes, with mucinous and undifferentiated cancers displaying the lowest and highest ratios, respectively (p = 0.005). The intratumoral TAM density significantly increased with increasing cancer stage and grade (p = 0.023 and 0.006, respectively). However, the overall M1/M2 TAM ratio decreased as the cancer stage increased (p = 0.012). In addition, the intra-islet M1/M2 ratio inversely correlated with the residual site size (p = 0.004). Among the TAM-related parameters, only the increased overall and intra-islet M1/M2 TAM ratios displayed prognostic significance in both the Kaplan-Meier survival and multivariate Cox regression analyses; however, the values of these two parameters did not differ significantly among the cancer histotypes.

**Conclusions:**

The patients with increased overall or intra-islet M1/M2 TAM ratios presented with an improved 5-year prognosis. Nevertheless, the TAM distribution patterns did not influence the overall outcomes of different ovarian cancer histotypes.

## Background

Ovarian cancer is highly malignant [1,2], and over 70% of ovarian cancer patients are diagnosed with severe peritoneal metastasis, with a 5-year survival rate of less than 30% [3,4]. As the cancer invades into the surrounding normal tissues, the monocyte-macrophage cellular system comprises a first line of defense [5,6]. Peripheral blood monocytes represent a major group of non-specific immune cells. These cells can perceive cancer invasion signals, migrate out of the capillaries, and transform into macrophages (immature, M0) [7-9]. Immature macrophages infiltrate into cancer islets or their surrounding tissues (i.e., the cancer stroma) and can subsequently be activated to recognize and kill genetically mutated cancer cells [10,11]. These macrophages are called tumor-associated macrophages (TAMs) [12]. However, not all TAMs fulfill their original tumoricidal potential. TAMs can be divided into two phenotypes with opposite functions [12-18]. The classically activated TAMs, the M1 phenotype, can be induced by interferon γ (IFNγ)/tumor necrosis factor α (TNFα) and exert a cytotoxic effect on cancer cells [12,13]. The alternatively activated TAMs, the M2 phenotype, can be induced by transforming growth factor β (TGFβ)/interleukin (IL) 4/IL13 and provide a nutritional advantage for cancer cells [12,14]. A critical difference between M1 and M2 TAMs is their secretion profiles [15]. M1 TAMs release reactive oxygen species, nitrogen intermediates, and inflammatory cytokines (e.g., IL1b, IL6, IL12, IL23, and TNF) that kill cancer cells; however, M2 TAMs release a variety of growth factors (e.g., epidermal growth factor [EGF], fibroblast growth factor [FGF], and vascular endothelial growth factor [VEGF]), that promote growth and vascularization of the cancer mass [15-18]. Cancer cells often secrete M2-type cytokines such as IL-10, CCL2/3/4/5/7/8, CXCL12, VEGF, and platelet-derived growth factor (PDGF) to recruit monocytes/M0 macrophages and direct them toward an M2 phenotype [19,20]. Therefore, M2 TAMs are frequently observed in cancer tissues [20].

A major characteristic of ovarian cancer is its heterogeneous nature [2,21-24]. According to the cells of origin, ovarian cancer can be classified into the following five histotypes: serous; mucinous; endometrioid; clear cell; and undifferentiated ovarian cancers [2]. These cancer histotypes possess different structural and cytological characteristics and confer different patient outcomes [2,25,26]. Generally, serous ovarian cancers are derived from the ovarian surface epithelium or fallopian tubes, and they represent the most frequently encountered clinical histotype [21-24]. Serous ovarian cancer patients have a poor to moderate 5-year outcome [25,26]. Mucinous ovarian cancer is derived from the transitional-type epithelium located at the tubal-mesothelial junction [22-24]. Patients with mucinous cancer typically have a relatively good prognosis [25,26]. Endometrioid and clear cell ovarian cancers originate from the endometrioid cyst of the ovary, and these cancer types are associated with a moderate to fair 5-year outcome [21-26]. Undifferentiated ovarian cancers are derived from the mutated basal/stem cells of the ovarian surface epithelium, such as the hilum cells [27]. Patients with this histotype always have a poor prognosis [27,28]. Previous studies demonstrated that TAMs have an important effect on cancer patient outcomes. For example, Edin et al. reported that increased infiltration of M1 macrophages at the cancer front is accompanied by a better prognosis in colorectal cancer patients [29], and Ohri et al. documented that an increased islet/stroma ratio of M1 TAMs is associated with a marked survival advantage for patients with non-small cell lung cancer [30]. However, the role of TAMs in ovarian cancer and their prognostic value remain unclear. Each ovarian cancer histotype exhibits a different protein expression profile [31,32]. Therefore, the cytokine profile produced by a certain ovarian cancer histotype might lead to a specific TAM distribution pattern (including cell density, differentiation, and microlocalization) in the cancer tissue. These unique TAM patterns could determine, at least in part, the clinical outcomes for ovarian cancer patients.

In this study, we aimed to address two core issues related to TAMs in ovarian cancer: (i) whether different histotypes of ovarian cancer are associated with different TAM distribution patterns and (ii) whether the altered TAM distribution patterns can confer different outcomes to ovarian cancer patients. Thus, the TAM data obtained from cancer specimens could be useful to comprehensively assess the long-term outcomes of ovarian cancer patients.

## Methods

### Study population

Consecutive ovarian cancer patients who were histopathologically diagnosed between January 1, 2002 and December 30, 2008 in the Department of Obstetrics and Gynecology, Ren Ji Hospital, School of Medicine, Shanghai Jiao Tong University, Shanghai, China, were enrolled in this study. Informed consent was obtained from all patients or their first-degree relatives. All enrolled patients had undergone cytoreductive surgery and a standardized postsurgical course of Taxol and platinum-based chemotherapy (i.e., the TP regimen). Paraffin-embedded cancer specimens from these patients were obtained from the clinical specimen bank of Renji Hospital. Patient medical histories were recorded including age, reproductive history, menopausal status, ascites, clinical stage (using the FIGO 2000 diagnostic system [33]), cancer histotype, grade, and 5-year follow-up outcome. The research protocol was approved by the ethics committee of Renji Hospital.

### Immunohistochemistry and immunofluorescence

Cancer specimens were sliced into 4-μm sections, de-waxed using xylene, and rehydrated with gradated ethanol. Antigen retrieval was performed using a microwave at > 90°C for 15 min, and the samples were allowed to cool to room temperature. The non-specific binding sites were blocked with 5% bovine serum albumin (BSA) for 1 h. For immunohistochemical staining, the sections were sequentially incubated with a mouse anti-human CD68 monoclonal antibody (clone KP1, Abcam, Cambridge, MA, USA; 1:100) and a horseradish peroxidase (HRP)-conjugated goat anti-mouse IgG antibody (Zhongshan, Beijing, China; 1:200). The antibody-binding sites were visualized using 3,3′-diaminobenzidine tetrahydrochloride (DAB; Zhongshan), and the cell nuclei were counterstained with hematoxylin. For immunofluorescent staining, the sections were incubated with a CF-488A (Biotium, San Francisco, CA, USA)-conjugated mouse anti-human CD68 polyclonal antibody (clone ZM0464, Zhongshan; 1:100) and one of the following CF-350 (Biotium)-conjugated antibodies at a 1:100 dilution: mouse anti-human HLA-DR monoclonal antibody (clone LN3, Zhongshan); rabbit anti-human iNOS monoclonal antibody (clone ZS6510, Zhongshan); mouse anti-human CD163 monoclonal antibody (clone 10D6, Zhongshan); or mouse anti-human VEGF monoclonal antibody (clone EP1176Y, Zhongshan). The nuclei were counterstained with SYTO 40 (Life Technologies, Grand Island, NY, USA). Two pathologists with no knowledge of the patient population independently reviewed the immunostained sections. Ten representative 400× fields were selected from each section, and only nucleated cells were analyzed. The obtained TAM densities were calculated as cells/mm^2^.

### Laser capture microdissection (LCM)-based flow cytometry

Paraffin-embedded cancer specimens were sliced into 15-μm sections and affixed onto FRAME slides (Leica, Wetzlar, Germany). LCM was performed based on the method described by Stany et al. [34]. Briefly, the sections were de-waxed, rehydrated, and stained with hematoxylin and eosin (H&E) to identify cancer islets and stromal regions. Approximately 20,000 cells/sample were collected from the selected specimen region using a LMD6500 Laser Microdissection system (Leica, Wetzlar, Germany). The obtained cells were re-suspended in phosphate-buffered saline (PBS) and centrifuged at 1,500 rpm for 3 min. For flow cytometry, cells were immunostained with a FITC-conjugated mouse anti-human CD68 monoclonal antibody (clone Ki-M7, Life Technologies; 1:200) and one of the following PE-conjugated antibodies: mouse anti-human keratin monoclonal antibody (clone C11, Abcam; 1:100); mouse anti-human iNOS monoclonal antibody (clone 4E5, Abcam; 1:150); mouse anti-human HLA-DR monoclonal antibody (clone L243, eBioscience, San Diego, California, USA; 1:100); mouse anti-human CD163 antibody (clone GHI/61, Affymetrix-eBioscience, San Diego, CA, USA; 1:200); or mouse anti-human VEGF monoclonal antibody (clone FLT-11, Sigma-Aldrich, St. Louis, MO, USA; 1:100). An FC500 MPL flow cytometer (Beckman Coulter, Brea, CA, USA) was used, and a total of 10,000 events were recorded for each flow cytometry run. The data are presented as the ratio of CD68^+^ TAMs (M1 or M2 TAMs) to the total number of cells analyzed by flow cytometry.

### Statistical analyses

A two-sided χ^2^ test was used to compare the demographic, clinical, and pathological characteristics (e.g., age, gravidity, parity, cancer stage, histotype, cancer grade, and size of residual site) of patient groups with a poor or extended survival time. ANOVA (or two-sided Student’s t test) was used to compare the TAM cell densities and differentiation patterns between patient groups with different levels of the described clinical-pathological characteristics. The Pearson’s product–moment correlation coefficient was used to estimate the relationship between the TAM (total, M1, and M2) densities determined through immunofluorescence and flow cytometry. Kaplan-Meier survival analysis was used to analyze the 5-year survival rates of patient groups with different TAM-related parameters. A multivariate Cox regression model was used to analyze the hazard ratios (HRs) of the clinical-pathological factors and the TAM-related parameters for patient survival and to determine their independence. SPSS 13.0 software was used for the analyses (IBM, Armonk, NY, USA), and p < 0.05 was considered significant.

## Results

### Patient characteristics and their relationships with TAM cell density, microlocalization, and differentiation patterns

**Figure 1:**
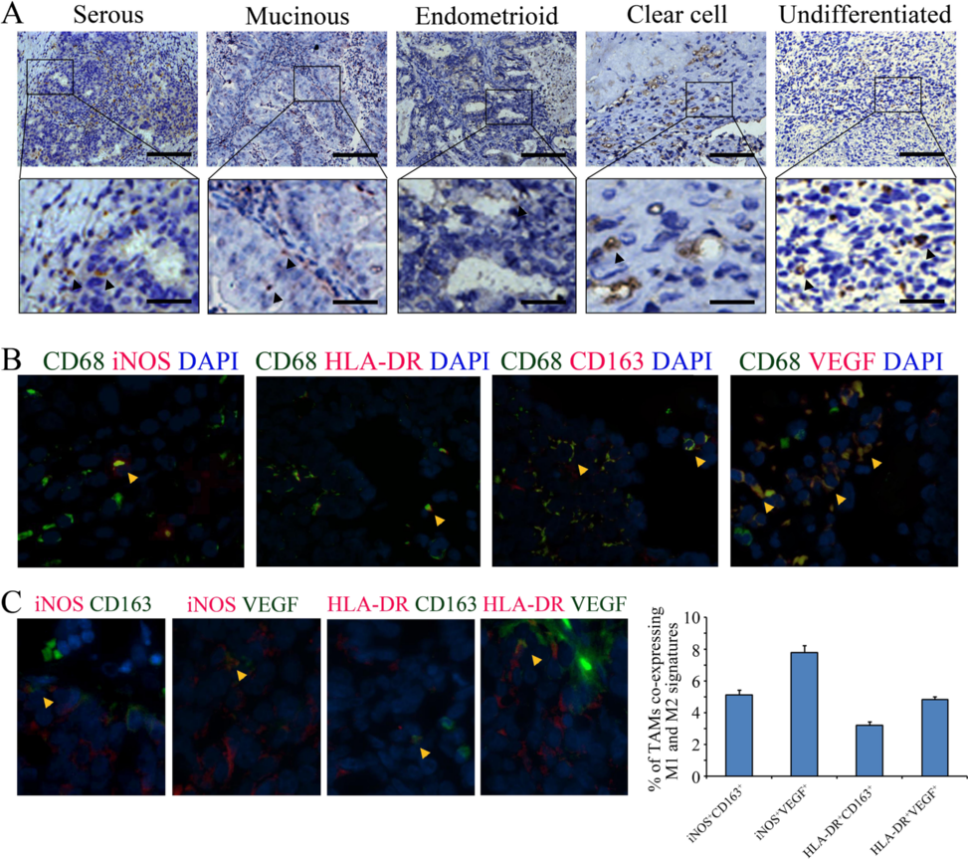
**Total, M1, and M2 TAMs immunostaining in ovarian cancer tissues. (A)** Immunohistochemical staining of CD68^+^ TAMs in various ovarian cancer histotypes. The arrowheads indicate CD68^+^ TAMs. The scale bars are 100 μm (upper row) and 25 μm (lower row). **(B)** Immunofluorescent staining of CD68^+^ TAMs expressing a single cellular differentiation biomarker (CD68^+^iNOS^+^/CD68^+^HLA-DR^+^ for M1 and CD68^+^CD163^+^/CD68^+^VEGF^+^ for M2) in ovarian cancer tissue. The arrowheads indicate M1 or M2 TAMs. **(C)** Immunofluorescent staining of TAMs co-expressing the M1 and M2 biomarkers in ovarian cancer tissue and the co-immunostaining percentages for different biomarker combinations in total TAMs. The arrowhead indicates TAMs that were co-immunostained with M1 and M2 biomarkers.

In total, 112 ovarian cancer patients were enrolled and divided into two groups according to their survival time: a poor survival group (survival time < 5 years) and an extended survival group (survival time ≥ 5 years). The patient demographic, clinical, and pathological characteristics are shown in Table 1. Then, the relationship between patient characteristics and TAM distribution patterns was analyzed (Table 2). To establish the TAM density in cancer tissues, the cells were immunohistochemically stained for CD68 (Figure 1A). To identify the TAM phenotypes, the cells were double-stained with fluorescent antibodies against CD68 and one of the two major M1 TAM biomarkers (iNOS or HLA-DR [30]) or one of the two major M2 TAM biomarkers (CD163 or VEGF [30]; Figure 1B). To determine whether an ambiguous TAM phenotypic classification might exist, we further assessed the co-expression patterns of M1 and M2 biomarkers in the same TAMs. We found that the percentages of iNOS + CD163 (5.1 ± 0.3%), iNOS + VEGF (7.8 ± 0.4%), and HLA-DR + VEGF (4.8 ± 0.2%) co-immunostained TAMs were significantly higher than the percentages (3.2 ± 0.2%) of HLA-DR + CD163 co-expressing cells (p < 0.001, ANOVA; Figure 1C). Therefore, HLA-DR and CD163 served as two major immunofluorescent identifiers of M1 and M2 TAMs in the following experiments.

**Table 1:** The demographic, clinical and pathological characteristics of the study population

**Characteristics**	**Poor survival group**	**Extended survival group**	**p value**
	(n = 63)*	(n = 49)*	
Age			0.196
< 40	5 (7.9)	3 (6.1)	
40 - 49	18 (28.6)	14 (28.6)	
50 - 59	21 (33.3)	11 (22.4)	
60 - 69	16 (25.4)	12 (24.5)	
≥ 70	3 (4.8)	9 (18.4)	
Gravidity			0.576
0 - 1	7 (11.1)	5 (10.2)	
2 - 3	29 (46.0)	19 (38.8)	
4 - 5	16 (25.4)	11 (22.4)	
≥ 5	11 (17.5)	14 (28.6)	
Parity			0.551
0 - 1	42 (66.7)	30 (61.2)	
2 - 3	21 (33.3)	19 (38.8)	
Menopause			0.789
Yes	37 (58.7)	30 (61.2)	
No	26 (41.3)	19 (38.8)	
Ascites			0.382
Yes	10 (15.9)	5 (10.2)	
No	53 (84.1)	44 (89.8)	
Peritoneal metastasis**			<0.001^#^
Yes	25 (39.7)	5 (10.2)	
No	38 (60.3)	44 (89.8)	
Lymphatic metastasis			0.024^#^
Yes	19 (30.2)	6 (12.2)	
No	44 (69.8)	43 (87.8)	
Stage			<0.001^#^
I	6 (9.5)	21 (42.9)	
II	21 (33.3)	20 (40.8)	
III	26 (41.3)	7 (14.3)	
IV	10 (15.9)	1 (2.0)	
Histotype			0.420
Serous	38 (60.3)	26 (53.1)	
Mucinous	5 (7.9)	9 (18.4)	
Endometrioid	9 (14.3)	8 (16.3)	
Clear cell	7 (11.1)	5 (10.2)	
Undifferentiated	4 (6.3)	1 (2.0)	
Grade			0.076
G1	9 (14.3)	15 (30.6)	
G2	28 (44.4)	21 (42.9)	
G3	26 (41.3)	13 (26.5)	
Size of residual site			0.007^#^
< 2 cm	43 (68.3)	44 (89.8)	
≥ 2 cm	20 (31.7)	5 (10.2)	

*The data are presented as the percentage.

**“Peritoneal metastasis” includes intestinal, bladder and liver metastasis of ovarian cancer and peritoneal lavage cytological test +.

^#^Statistical significance, two-sided χ^2^ test.

**Table 2:** Intratumoral TAM densities and differentiation patterns in relation to clinical and pathological characteristics

**Characteristics**	Total TAM densities*			M1 TAM densities*			M2 TAM densities*		
	**Overall**	**Islet**	**Stroma**	**Overall**	**Islet**	**Stroma**	**Overall**	**Islet**	**Stroma**
Stage	*(p = 0.023)*^#^	*(p = 0.206)*	*(p = 0.004)*^#^	*(p = 0.162)*	*(p = 0.828)*	*(p = 0.038)*^#^	*(p = 0.002)*^#^	*(p = 0.008)*^#^	*(p = 0.002)*^#^
I	30.2 ± 18.9	16.3 ± 12.1	14.0 ± 8.5	17.6 ± 12.1	10.2 ± 8.0	7.4 ± 5.2	12.7 ± 7.6	6.1 ± 4.5	6.6 ± 4.1
II	36.2 ± 23.7	17.0 ± 11.1	19.2 ± 14.0	21.5 ± 14.0	11.3 ± 8.3	10.2 ± 7.6	14.7 ± 10.9	5.7 ± 3.4	9.0 ± 8.3
III	49.5 ± 32.6	22.4 ± 14.7	27.0 ± 19.5	26.8 ± 19.0	12.3 ± 8.8	14.4 ± 13.3	22.7 ± 16.3	10.1 ± 9.1	12.6 ± 8.4
IV	52.7 ± 47.7	22.4 ± 21.6	30.4 ± 24.5	23.5 ± 18.8	11.2 ± 12.3	12.4 ± 9.9	29.2 ± 28.4	11.2 ± 10.6	18.0 ± 18.7
Histotype	*(p = 0.049)*^#^	*(p = 0.038)*^#^	*(p = 0.015)*^#^	*(p = 0.016)*^#^	*(p = 0.047)*^#^	*(p = 0.006)*^#^	*(p = 0.193)*	*(p = 0.150)*	*(p = 0.170)*
Serous	45.9 ± 33.9	22.3 ± 16.0	23.6 ± 18.1	25.1 ± 16.4	13.3 ± 9.8	11.9 ± 9.7	20.8 ± 18.5	9.1 ± 8.4	11.7 ± 11.3
Mucinous	45.1 ± 28.2	15.1 ± 9.0	30.0 ± 20.1	27.3 ± 19.8	9.8 ± 6.1	17.5 ± 14.2	17.8 ± 9.8	5.3 ± 3.1	12.5 ± 7.5
Endometrioid	26.9 ± 15.3	13.7 ± 8.4	13.2 ± 7.7	14.9 ± 10.1	8.1 ± 5.9	6.8 ± 5.1	12.1 ± 6.8	5.6 ± 3.4	6.4 ± 3.9
Clear cell	26.2 ± 12.0	12.6 ± 4.4	13.6 ± 7.9	12.3 ± 5.0	6.8 ± 3.9	5.6 ± 2.7	13.8 ± 9.6	5.8 ± 4.2	8.0 ± 6.5
Undifferentiated	34.8 ± 22.6	19.8 ± 13.3	15.0 ± 10.3	21.8 ± 16.0	13.2 ± 10.9	8.6 ± 5.9	13.0 ± 6.9	6.6 ± 2.9	6.4 ± 4.7
Grade	*(p = 0.001)*^#^	*(p < 0.001)*^#^	*(p = 0.002)*^#^	*(p < 0.001)*^#^	*(p = 0.002)*^#^	*(p = 0.002)*^#^	*(p = 0.006)*^#^	*(p = 0.003)*^#^	*(p = 0.028)*^#^
G1	30.2 ± 18.2	13.0 ± 8.8	17.2 ± 12.3	17.6 ± 11.9	8.2 ± 6.2	9.4 ± 7.7	12.6 ± 7.7	4.8 ± 3.3	7.8 ± 5.9
G2	34.1 ± 20.6	16.7 ± 9.8	17.4 ± 12.0	18.2 ± 11.5	9.9 ± 6.8	8.3 ± 6.0	15.9 ± 11.1	6.8 ± 4.1	9.1 ± 7.9
G3	54.4 ± 37.5	25.5 ± 17.8	28.9 ± 21.6	30.4 ± 19.6	15.1 ± 10.8	15.3 ± 12.8	24.0 ± 20.8	10.4 ± 9.9	13.6 ± 12.3
Size of residual site	*(p = 0.625)*	*(p = 0.650)*	*(p = 0.637)*	*(p = 0.963)*	*(p = 0.497)*	*(p = 0.595)*	*(p = 0.328)*	*(p = 0.077)*	*(p = 0.773)*
< 2 cm	39.6 ± 29.6	18.6 ± 13.8	20.9 ± 17.4	22.4 ± 16.0	11.6 ± 8.4	10.7 ± 10.1	17.2 ± 15.6	7.0 ± 6.5	10.2 ± 10.1
≥ 2 cm	42.8 ± 27.4	20.0 ± 14.1	22.7 ± 14.9	22.2 ± 15.7	10.3 ± 8.8	11.9 ± 8.6	20.6 ± 14.3	9.7 ± 8.0	10.8 ± 7.8
**Characteristics**	Islet/stroma ratio of*			**Overall ratio of**		**Intra-islet ratio of**		**Intra-stroma ratio of**	
	**Total TAMs**	**M1 TAMs**	**M2 TAMs**	M1/M2 TAMs*		M1/M2 TAMs*		M1/M2 TAMs*	
Stage	*(p = 0.054)*	*(p = 0.585)*	*(p = 0.406)*	*(p = 0.012)*^#^		*(p = 0.106)*		*(p = 0.490)*	
I	1.3 ± 0.7	1.7 ± 1.2	1.1 ± 0.8	1.4± 0.5		1.8 ± 0.8		1.2 ± 0.7	
II	1.1 ± 0.6	1.5 ± 1.3	0.9 ± 0.7	1.5± 0.5		2.0 ± 0.9		1.4 ± 0.7	
III	0.9 ± 0.3	1.2 ± 0.9	0.8 ± 0.4	1.3± 0.6		1.6 ± 0.9		1.2 ± 0.7	
IV	0.9 ± 0.6	1.6 ± 2.6	1.0 ± 0.9	1.0± 0.5		1.3 ± 1.0		1.1 ± 0.7	
Histotype	*(p = 0.005)*^#^	*(p = 0.197)*	*(p = 0.073)*	*(p =0.129)*		*(p = 0.651)*		*(p = 0.377)*	
Serous	1.1 ± 0.6	1.6 ± 1.2	1.0 ± 0.7	1.4± 0.5		1.8 ± 0.9		1.3 ± 0.7	
Mucinous	0.6 ± 0.2	0.6 ± 0.2	0.5 ± 0.3	1.5± 0.5		1.9 ± 0.6		1.5 ± 0.7	
Endometrioid	1.1 ± 0.6	1.6 ± 2.1	1.0 ± 0.3	1.3± 0.2		1.5 ± 0.9		1.2 ± 0.7	
Clear cell	1.0 ± 0.3	1.6 ± 1.3	1.0 ± 0.5	1.1± 0.5		1.6 ± 1.2		1.0 ± 0.6	
Undifferentiated	1.5 ± 0.8	1.6 ± 0.8	1.4 ± 1.0	1.6± 0.5		1.9 ± 0.9		1.5 ± 0.5	
Grade	*(p = 0.671)*	*(p = 0.228)*	*(p = 0.925)*	*(p =0.159)*		*(p = 0.504)*		*(p = 0.231)*	
G1	1.0 ± 0.7	1.2 ± 0.8	0.9 ± 0.9	1.5± 0.6		1.8 ± 0.8		1.4 ± 0.7	
G2	1.1 ± 0.7	1.7 ± 1.7	1.0 ± 0.6	1.3 ± 0.5		1.6 ± 0.8		1.1 ± 0.6	
G3	1.0 ± 0.5	1.3 ± 1.1	0.9 ± 0.6	1.4± 0.6		1.8 ± 1.0		1.3 ± 0.7	
Size of residual site	*(p = 0.816)*	*(p = 0.533)*	*(p = 0.025)*^#^	*(p =0.059)*		*(p =0.004)*^#^		*(p = 0.689)*	
< 2 cm	1.0 ± 0.5	1.5 ± 1.2	0.9 ± 0.6	1.4± 0.5		1.9 ± 0.9		1.2 ± 0.7	
≥ 2 cm	1.1 ± 0.7	1.3 ± 1.7	1.2 ± 0.8	1.2± 0.6		1.3 ± 0.9		1.3 ± 0.7	

*The data are represented as mean ± standard deviation.

^#^Statistical significance, ANOVA.

The obtained immunostaining results indicated that CD68^+^ TAM density was highest in serous ovarian cancer followed by mucinous, undifferentiated, endometrioid, and clear cell cancers (p = 0.049, ANOVA; Table 2). Within the cancer tissue, the intra-islet TAM density was highest in serous cancer followed by undifferentiated, mucinous, endometrioid, and clear cell cancers (p = 0.038, ANOVA; Table 2). Significantly increased intra-stromal TAM densities were observed in mucinous and serous cancers (p = 0.015, ANOVA; Table 2). TAM densities (total, intra-islet, and intra-stromal) were significantly increased with increasing clinical stage (except for the intra-islet TAMs, p = 0.023 and p = 0.004, respectively, ANOVA) and pathological grade (p = 0.001, p < 0.001, and p = 0.002, respectively, ANOVA; Table 2). The islet/stroma ratio of total TAMs varied among the cancer histotypes, with mucinous and undifferentiated cancer displaying the lowest and highest ratios, respectively (p = 0.005, ANOVA; Table 2). Nevertheless, these ratios did not correlate with cancer stage and grade (Table 2). For M1 TAMs, the total, intra-islet, and intra-stromal cell densities were significantly different between the cancer histotypes, with mucinous cancer having the highest total (islet + stroma) and intra-stromal densities (p = 0.016 and p = 0.047, respectively, ANOVA) and serous cancer possessing the highest intra-islet cell density (p = 0.006, ANOVA; Table 2). The islet/stroma M1 TAM ratios did not differ between cancer histotypes (Table 2). For M2 TAMs, the total, intra-islet, and intra-stromal cell densities increased with increasing cancer stage (p = 0.002, p = 0.008 and p = 0.002, ANOVA), and a significant decreasing trend in the overall M1/M2 TAM ratio was observed in cancer specimens from Stage I through Stage IV (p = 0.012, ANOVA; Table 2). Both M1 and M2 TAM cell densities (total, intra-islet, and intra-stroma) significantly increased with cancer grade (p < 0.001, p = 0.002, and p = 0.002 for M1; p = 0.006, p = 0.003, and p = 0.028 for M2; ANOVA); however, the overall, intra-islet, and intra-stromal M1/M2 TAM ratios remained relatively stable among the different cancer grades (Table 2). Moreover, the size of the residual site was positively associated with the islet/stroma ratio of M2 TAMs (p = 0.025, ANOVA) and inversely correlated with the intra-islet M1/M2 TAM ratio (p = 0.004, ANOVA; Table 2).

### LCM-based flow cytometry validation of the immunofluorescence analysis on TAMs

**Figure 2:**
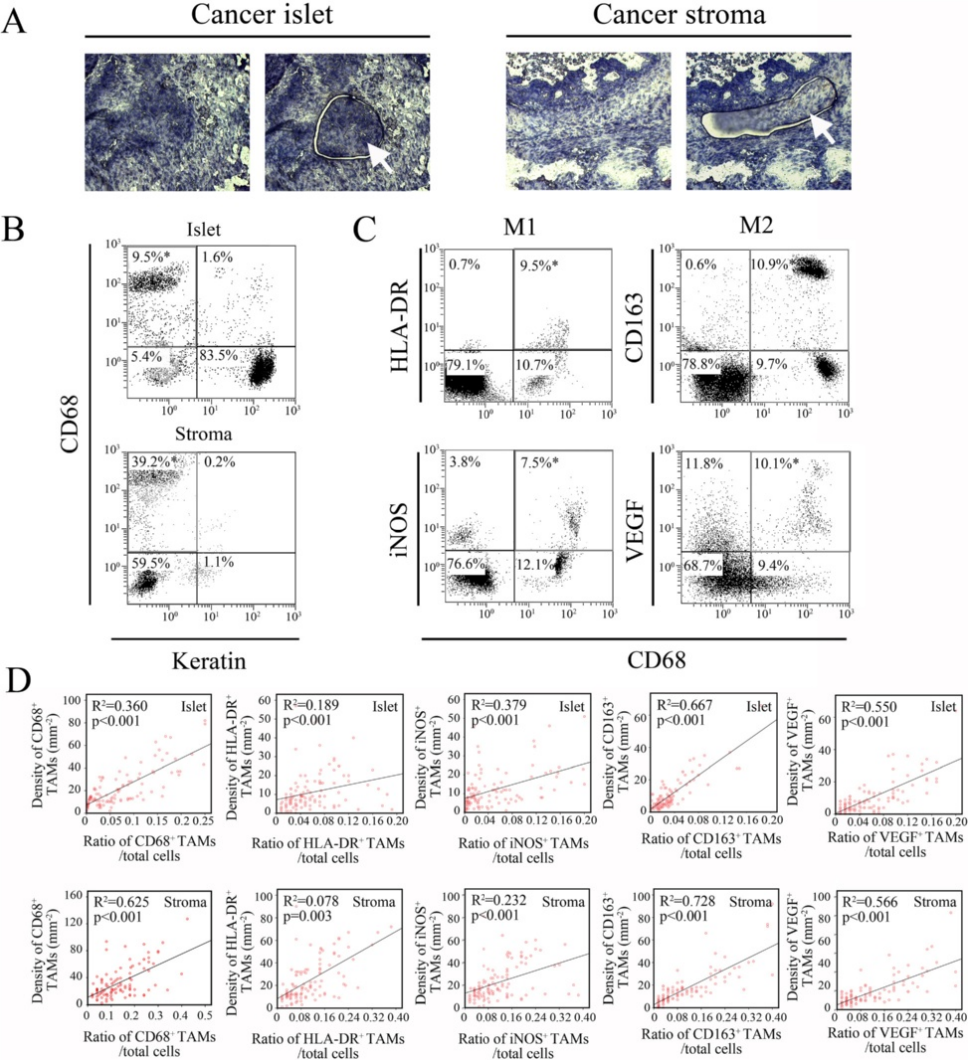
**Flow cytometric validation of immunofluorescence analysis of total, M1, and M2 TAMs in ovarian cancer. (A)** Cancer islet and stromal regions were microdissected from tissue sections. The obtained tissues were dissociated into cell suspensions and subjected to flow cytometry analysis. The arrows indicate the microdissected cancer tissue. **(B)** Representative flow cytometry analyses of the cell suspensions obtained from the cancer islet and stromal regions. The cells were stained with PE-conjugated anti-keratin and FITC-conjugated anti-CD68 monoclonal antibodies. The asterisk indicates the percentage of CD68^+^ TAMs (keratin^-^) in the total cells, as determined through flow cytometry. **(C)** Representative flow cytometry analyses of M1 and M2 TAMs in a cell suspension. The cells were stained with PE-conjugated anti-HLA-DR (or anti-CD163, anti-iNOS, and anti-VEGF) and FITC-conjugated anti-CD68 monoclonal antibodies. The asterisk indicates the percentage of M1 or M2 TAMs in the total cell population, as determined through flow cytometry. **(D)** The correlations between the total, M1, and M2 TAM densities, as determined through immunofluorescence and the TAM ratios in the total cells analyzed through flow cytometry. R^2^ indicates the square of the Pearson’s product–moment correlation coefficient.

The tissue sections subjected to immunohistochemistry and immunofluorescence were re-examined by flow cytometry to validate the pathologists’ counting results of TAMs (total, M1, and M2). To differentiate between the intra-islet and intra-stromal TAMs, we laser microdissected the islet and stromal regions from each cancer tissue section (Figure 2A). The percentages of total, intra-islet, and intra-stromal TAMs as well as M1 and M2 TAMs were assessed through flow cytometry using cell suspensions obtained from paraffin-embedded specimens (Figure 2B and C). Flow cytometry analysis revealed a notable linear relationship between the ratios of interested (ie. total, M1 and M2) TAMs/total cells and the TAM densities defined by immunofluorescent analysis (Figure 2D), confirming that the two-pathologist section-reviewing system is a reliable method for TAM analysis in ovarian cancer.

### Kaplan–Meier survival analysis based on the intratumoral distribution patterns of TAMs

**Figure 3:**
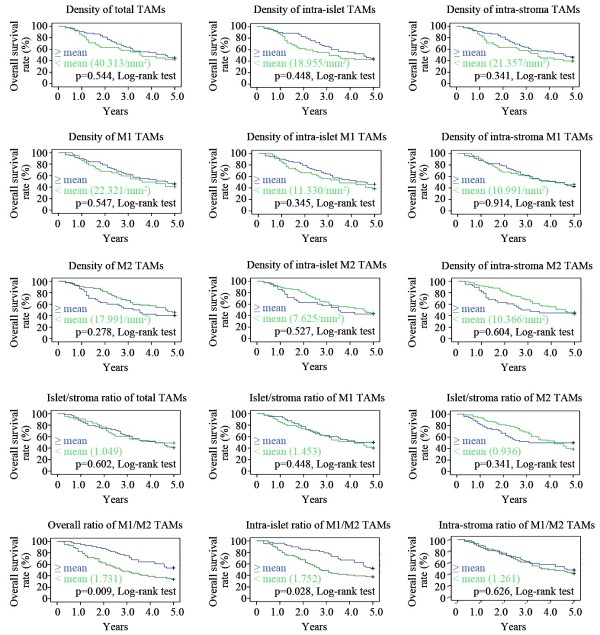
**Kaplan-Meier survival analyses of TAM-related parameters in ovarian cancer patients.** The overall 5-year survival rates were compared between patient groups (less than the mean vs. greater than the mean) for each TAM-related parameter. Only two parameters, the overall ratio of M1/M2 TAMs and the intra-islet ratio of M1/M2 TAMs, revealed significant differences between the two patient groups.

To explore the prognostic effects of different TAM distribution patterns on the 5-year survival rate of ovarian cancer patients, we performed Kaplan-Meier survival analyses to determine the densities of the following groups: total, intra-islet, and intra-stromal TAMs; total, intra-islet, and intra-stromal densities of M1 and M2 TAMs; islet/stromal ratios of total, M1, and M2 TAMs; and overall, intra-islet and intra-stromal M1/M2 TAM ratios. Only the overall and intra-islet M1/M2 TAM ratios displayed significant prognostic effects on patient survival (p = 0.009 and p = 0.028, respectively, log-rank test; Figure 3). The 5-year survival rates were 53.6% and 52.1% above the means for the overall and intra-islet M1/M2 TAM ratios, respectively, compared with 33.9% and 37.5% for these parameters below the means (Figure 3).

### Multivariate Cox regression analysis of the independence of TAM-related parameters

Kaplan-Meier analysis may be affected by confounding factors so that its results cannot reflect the actual effect of each individual TAM characteristic. Therefore, we performed multivariate Cox regression analysis on the independent prognostic values of TAM-related parameters. The TAM distribution patterns in the “poor survival” and “extended survival” groups are listed in Table 3. The reference parameters used in the multivariate analysis included patient age, gravidity, parity, ascites, peritoneal metastasis, lymphatic metastasis, size of residual site, clinical stage, cancer histotype, and pathological grade (Table 4). For each Cox regression analysis, one TAM parameter was evaluated. We found that M1 TAM densities (total and intra-islet) and M1/M2 TAM ratios (overall and intra-islet) displayed independent effects on patient outcome (Table 4 and Additional file 1: Tables S1-S14). Of these parameters, the overall M1/M2 TAM ratio (≥ 1.371) exhibited the lowest HR for patients (0.448, 95% confidence interval [CI]: 0.238-0.842; Table 3) followed by the intra-islet M1/M2 ratio (ratio ≥ 1.752, HR = 0.510, 95% CI: 0.264-0.986; Additional file 1: Table S13).

**Table 3:** Intratumoral TAM densities and differentiation patterns in relation to patient survival time

**Patient groups**	Total TAM densities*			M1 TAM densities*			M2 TAM densities*		
	**Overall**	**Islet**	**Stroma**	**Overall**	**Islet**	**Stroma**	**Overall**	**Islet**	**Stroma**
Poor survival group	41.7 ± 30.1	19.4 ± 14.3	22.3 ± 16.9	22.0 ± 15.0	10.8 ± 8.1	11.2 ± 9.5	19.6 ± 17.5	8.5 ± 8.4	11.1 ± 10.3
Extended survival group	38.6 ± 27.8	18.4 ± 13.3	20.2 ± 16.8	22.7 ± 17.1	12.0 ± 9.5	10.8 ± 10.1	15.9 ± 11.8	6.4 ± 4.1	9.4 ± 8.7
p value^#^	0.583	0.713	0.518	0.535	0.819	0.503	0.822	0.761	0.199
**Patient groups**	Islet/stroma ratio of*			**Overall ratio of**		**Intra-islet ratio of**		**Intra-stroma ratio of**	
	**Total TAMs**	**M1 TAMs**	**M2 TAMs**	M1/M2 TAMs*		M1/M2 TAMs*		M1/M2 TAMs*	
Poor survival group	1.0 ± 0.6	1.5 ± 1.5	0.9 ± 0.7	1.3 ± 0.6		1.7 ± 1.0		1.2 ± 0.7	
Extended survival group	1.1 ± 0.6	1.4 ± 1.1	0.9 ± 0.6	1.5 ± 0.5		1.8 ± 0.7		1.4 ± 0.7	
p value	0.113	0.364	0.930	0.061		0.802		0.225	

*The data are represented as mean ± standard deviation.

^#^Statistical significance, two-sided Student’s t test.

**Table 4:** Multivariate Cox regression analysis of clinical, pathological, and TAM-related prognostic factors for ovarian cancer*

**Parameters**	**HR**	**95% CI**	**p value**
Age			0.234
< 40	1 (reference)	-	
40 - 49	1.016	(0.205, 4.931)	
50 - 59	1.664	(0.386, 7.181)	
60 - 69	1.239	(0.292, 5.253)	
≥ 70	0.329	(0.043, 2.492)	
Gravidity			0.726
0 - 1	1 (reference)	-	
2 - 3	1.000	(0.424, 2.355)	
4 - 5	0.904	(0.345, 2.367)	
≥ 5	0.612	(0.184, 2.031)	
Parity			0.851
0 - 1	1 (reference)	-	
2 - 3	1.068	(0.536, 2.129)	
Menopause			0.944
Yes	1 (reference)	-	
No	0.969	(0.407, 2.304)	
Ascites			0.120
Yes	1 (reference)	-	
No	0.421	(0.142, 1.252)	
Peritoneal metastasis			<0.001^#^
Yes	1 (reference)	-	
No	10.753	(3.768, 30.685)	
Lymphatic metastasis			0.678
Yes	1 (reference)	-	
No	0.814	(0.307, 2.156)	
Stage			<0.001^#^
I	1 (reference)	-	
II	3.5	(0.781, 15.677)	
III	6.1	(1.733, 21.469)	
IV	38.5	(7.377, 200.926)	
Histotype			0.003^#^
Serous	1 (reference)	-	
Mucinous	0.119	(0.017, 0.855)	
Endometrioid	0.560	(0.116, 2.707)	
Clear cell	0.780	(0.146, 4.159)	
Undifferentiated	5.952	(1.339, 26.451)	
Grade			0.002^#^
G1	1 (reference)	-	
G2	4.557	(2.373, 8.753)	
G3	8.197	(2.456, 27.362)	
Size of residual site			0.008^#^
< 2 cm	1 (reference)	-	
≥ 2 cm	2.786	(1.307, 5.937)	
Overall M1/M2 TAM ratio			0.013^#^
< 1.371	1 (reference)	-	
≥ 1.371	0.448	(0.238, 0.842)	

*A representative table for multivariate Cox regression analysis is shown for TAM-related parameters (i.e., overall M1/M2 TAM ratio). For the HRs of other TAM-related parameters, see Additional file 1: Tables S1-S14.

^#^Statistical significance.

### Prognostic effects of overall and intra-islet M1/M2 TAM ratios in ovarian cancer histotypes

**Figure 4:**
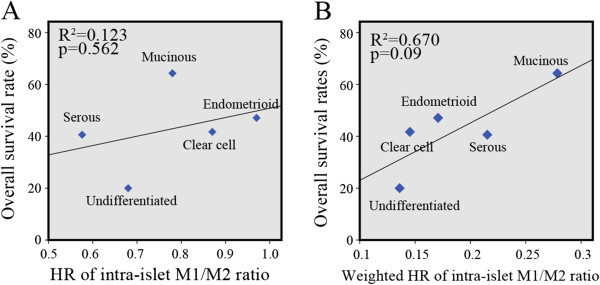
**The relationship between intra-islet M1/M2 ratio HRs and the 5-year survival of different histotypes. (A)** The HRs of the five ovarian cancer histotypes were compared with the corresponding 5-year survival rates of each histotype. **(B)** The HRs were weighted with the ratios of their affected population in each histotype group, and the groups were further compared with the 5-year survival rates of the corresponding cancer histotypes. R^2^ indicates the square of the Pearson’s product–moment correlation coefficient.

The prognostic effects of the overall and intra-islet M1/M2 TAM ratios for patients with serous (64 cases), mucinous (14 cases), endometrioid (17 cases), clear cell (12 cases), and undifferentiated (5 cases) ovarian cancers were further evaluated. The mean overall and intra-islet M1/M2 ratios were calculated for each ovarian cancer histotype (Table 5). Multivariate Cox regression analyses indicated that the HRs for the overall M1/M2 ratio lost their significance in three of the five histotypes analyzed, and the HRs of the intra-islet M1/M2 ratio were the lowest (0.576, 95% CI: 0.333-0.996) in the serous histotype and highest (0.97, 95% CI: 0.912-0.991) in the endometrioid histotype (Table 5). We also analyzed the relationship between the histotype-specific HRs of the intra-islet M1/M2 ratio and the corresponding 5-year survival rates for each cancer histotype (Figure 4). To reflect the actual hazards of the intra-islet M1/M2 ratio on enrolled patients, the HRs were further multiplied by the ratio patients presenting an intra-islet M1/M2 ratio greater than the mean (because the analyzed HRs were only significant in these patients; Table 5) for each histotype (Figure 4). However, no significant correlations were observed using either approach, indicating that the intra-islet M1/M2 ratio does not contribute to the different outcomes of ovarian cancer histotypes.

**Table 5:** Ovarian cancer histotype-specific HRs of overall and intra-islet M1/M2 TAM ratios*

**Ovarian cancer histotypes**	**Overall M1/M2 TAM ratio**				**Intra-islet M1/M2 TAM ratio**			
	**Mean**	**HR**	**95% CI**	**p value**	**Mean**	**HR**	**95% CI**	**p value**
Serous	1.395	0.560	(0.244, 1.286)	0.172	1.802	0.576	(0.333, 0.996)	0.048^#^
Mucinous	1.550	0.756	(0.465, 0.937)	0.046^#^	1.909	0.783	(0.552, 0.973)	0.023^#^
Endometrioid	1.264	0.923	(0.913, 0.996)	0.049^#^	1.480	0.978	(0.925, 0.997)	0.035^#^
Clear cell	1.074	0.856	(0.485, 0.993)	0.112	1.637	0.875	(0.715, 0.982)	0.042^#^
Undifferentiated	1.632	0.712	(0.587, 0.926)	0.074	1.884	0.681	(0.467, 0.935)	0.038^#^

*HRs represent the multivariate Cox regression analysis results for the patient groups with an overall or intra-islet M1/M2 TAMs ratio greater than the mean. The patient groups with an overall or intra-islet M1/M2 TAMs ratio less than the mean were set as reference groups (HR = 1). To be concise, the reference groups were omitted from the table.

^#^Statistical significance.

## Discussion

The role of TAMs in cancer progression has been investigated in many types of human malignancies, including lung cancer, oral squamous cell carcinoma, esophageal cancer, gastric cancer, pancreatic cancer, liver cancer, intrahepatic cholangiocarcinoma, colorectal cancer, thyroid cancer, breast cancer, endometrial cancer, cervical cancer, bladder cancer, and prostate cancer [29,30,35-47]. The majority of these studies indicated that TAM infiltration density, differentiation status, and microlocalization influence the 5-year patient outcome. To our knowledge, the current study represents the most comprehensive analysis of TAMs in ovarian cancer based on a large-scale study population. Moreover, we compared the different histotypes of ovarian cancer, illustrating the unique pathological effects of cancer histotypes on TAM characteristics. This study enhances our understanding of TAMs and contributes to the growing data regarding the prognostic value of TAMs in clinical oncology.

Our data demonstrated that serous, mucinous, and undifferentiated ovarian cancer histotypes most frequently display TAM infiltration. Among these histotypes, a subtle difference existed; for serous and undifferentiated ovarian cancers, the TAMs gathered in the cancer islet, whereas for mucinous ovarian cancer, the TAMs accumulated in the stromal region (Table 2). There are several possible explanations for these phenomena. First, serous and mucinous cancers often express an abundant amount of mucins such as Mucin 1 (i.e., CA153), Mucin 2, Mucin 4, and Mucin 16 (i.e., CA125) [48-54]. Some mucins, such as Mucin 2 and Mucin 16, are immunomodulatory factors that can increase the expression of chemoattractants or pro-inflammatory factors including MCP-1, IL8, and PGE2 [50,54,55] in mucin-secreting cancer cells; therefore, more monocytes/macrophages can be recruited into the local tissue. In contrast, endometrioid and clear cell cancers express a relatively low amount of these mucins [48,52,56], which is associated with significantly lower TAM densities in the cancer tissue. A mucin-based theory can partially explain the different TAM infiltration densities observed between the serous/mucinous and endometrioid/clear cell ovarian cancer tissues. However, for the undifferentiated ovarian cancer histotype, we inferred that because of its primordial nature, more embryonic proteins could be synthesized and released [57], which could lead to a more severe specific/non-specific immune response in the local tissue [58]. Therefore, an increased number of TAMs were present. To answer the question why the TAM microlocalization patterns (including the total and M1 TAMs) were significantly different between ovarian cancer histotypes (Table 2), we consider these differences might be attributed to unique gene expression profiles and cytological behaviors of the cancer cells. For example, the peri-cancer-islet mucin layers built by serous and mucinous cancer cells are quite different. Serous cancer cells secrete Mucin 16 as the major mucin molecule, which is almost soluble in serum [59,60]. The mucin layer surrounding the serous cancer islet is rather thin and dispersed [59,60]. Mucinous cancer cells secrete Mucin 2 and 5 as their major mucin forms, which create a condensed and gel-like layer surrounding the cancer cells [56,59,61-63]. This heavy layer can obstruct TAM access to the inside of the mucinous cancer islet. Meanwhile, the infiltration contribution of M1 TAMs was more significant than that of M2 TAMs for achieving the histotype-specific microlocalization patterns of intratumoral TAMs (Table 2), suggesting that the chemotactic effect of mucins may be more potent for M1 TAMs. Undifferentiated cancer cells displayed a highly disorganized tissue structure, with the cancer islet and stromal regions interwoven; therefore, more TAMs could obtain access to the cancer islets. Interestingly, these increased intra-islet TAMs were also predominantly M1 TAMs (Table 2), indicating that chemoattractants secreted by undifferentiated cancer cells are more effective at attracting M1 TAMs compared with M2 TAMs. Certainly, the molecular mechanisms discussed here require more detailed investigations, and currently, the histotype-dependent TAM distribution patterns suggest that TAMs could have diverse effects on the growth and development of different ovarian cancer histotypes.

Moreover, our observations indicated that the infiltrating behavior of TAMs was associated with the pathological grade of ovarian cancer (Table 2). Of all histotypes of ovarian cancer, the densities of total, M1, and M2 TAMs were the highest in G3 cases, followed by G2 and G1 cases. Several research groups have reported this phenomenon in other human malignancies. For example, Medrek et al. demonstrated that breast cancer cases with a dense infiltration of macrophages were mostly of a higher pathological grade [34]. Komohara et al. observed that the number of intratumoral microglia/macrophages correlated with the pathological grade of gliomas [64]. However, in colorectal cancer, Edin et al. did not observe a correlation between TAM density and pathological grade [29]. Thus, the relationship between TAM density and cancer pathological grade is dependent on the exact anatomic site of the cancer. Based on our observations, it is possible that the TAM density/pathological grade relationship was due to the disorganized/discontinuous tissue structure of the higher pathological graded ovarian cancers, which could allow peripheral-blood monocytes/macrophages (M1 or M2 TAMs) easy entry into the cancer tissue bed [33]. Alternatively, we cannot discount the possibility that high-grade ovarian cancer cells could release more chemoattractants or pro-inflammatory factors such as chromatin-binding protein high mobility group B1 (HMGB1) [65] and calreticulin (CRT) [66] into the surrounding tissues to attract more monocytes/microphages to the cancer tissue [67]. However, in addition to the changes in TAM densities, we did not observe any changes in TAM microlocalization (islet vs. stroma) or differentiation (M1 vs. M2) between the different ovarian cancer grades; therefore, the exact clinical significance of varying TAM densities likely resides in the intrinsic properties of these infiltrating TAMs, which cannot be affected by the cancer grade.

The relationship between cancer stage and TAM density has reached significance in the patient population enrolled by our study, suggesting that the clinical stage could potentially influence TAM infiltration (Table 2). Ovarian cancer mainly metastasizes via peritoneal dissemination and implantation [1-4]. The omentum is the most frequent site for ovarian cancer metastasis [1-4]. The peritoneal cavity is rich in “resident” tissue macrophages, which are widely distributed along the surface of the peritoneum and omentum [5,12]. In the study, the increased TAM densities (overall, intra-islet, and intra-stromal) were found to be correlated with the degree of the cancer tissue invasion into the peritoneal cavity and organs. Hence, the peritoneal macrophages are probably the primary source responsible for vigorous TAM infiltration observed in ovarian cancer tissues with higher clinical stages. Furthermore, as reported in previous studies, the innate peritoneal macrophage defensive barrier can play an important role in delaying cancer progression [68] or contribute to the formation of malignant ascites and/or promote severe peritoneal metastasis in ovarian cancer patients [69]. Such outcomes largely depend on the dominant phenotype of macrophages infiltrating the cancer tissue [68-70]. Here, we observed that the M2 TAM densities (overall, intra-islet, and intra-stromal) consistently increased with clinical stage, while the corresponding M1/M2 ratios declined as the cancer stage increased (Table 2). Also, the islet/stroma ratio of M2 TAMs was increased in ≥ 2 cm residual sites; and the intra-islet M1/M2 TAM ratio was decreased in these residual sites (Table 2). Considering that the clinical stage (or the size of residual site) is apparently not a TAM differentiation-inducing factor, these phenomena suggest that the increased M2 TAM infiltration could have promoted the aggressive behavior in ovarian cancer cells, which favors their widespread metastasis in the peritoneal cavity.

With regard to the long-term prognostic value of TAMs in ovarian cancer patients, there have been three major immune-microenvironment theories: the TAM density theory [29]; the M1-dominant theory [71]; and the M2-dominant theory [40]. The TAM density theory supposes a differentiation-independent role for TAMs in the outcome of cancer patients, while the latter two emphasize the differentiation status-dependent roles of TAMs in the 5-year survival. The data obtained by our study both supports and conflicts with these theories, which reflects the unique effects of TAMs in ovarian cancer. All these consistencies and inconsistencies require a more thorough analysis to provide an in-depth understanding of the clinical significance of TAMs.

For the density theory, a number of early studies indicated that a high density of intratumoral TAMs correlates with reduced patient survival, regardless of the TAM phenotypes [43,72,73]. Moreover, results from animal models that used macrophage-depleted mice to explore the role of TAMs in cancer progression agreed with these clinical findings [74,75]. However, in recent years, increasing evidence indicates a different effect of TAMs on patient prognosis. A representative study of 446 colorectal cancer specimens by Forssell et al. indicated that intensive macrophage infiltration at the cancer front is associated with an improved prognosis [76]. Furthermore, Edin et al. indicated that a simultaneous increase in M1 and M2 TAM densities at the invasive front of colorectal cancer predicts a positive outcome [29]. Ohri et al. obtained similar observations in non-small cell lung cancer (NSCLC), and they speculated that M2 TAMs might exert the same effect as M1 TAMs in cancer islets; otherwise, their observations could not be explained [30]. Our current study analyzed the prognostic value of TAM density based on four TAM-related parameters, namely, the densities of the overall, intra-islet, and intra-stromal TAMs and the islet/stroma TAM ratio. However, none of the four parameters correlated with patient prognosis in either Kaplan-Meier survival or multivariate Cox regression analyses. These results suggest that increased TAM infiltration alone, even when present in the cancer islet, cannot provide as powerful of a tumoricidal effect in ovarian cancer compared with that observed in colorectal or lung cancer. There are several probable reasons for the difference in these effects. One potential explanation is that ovarian cancer cells of any histotype might be less sensitive or more resistant to TAM attacks than colorectal or lung cancer cells. Unfortunately, there is little in vivo or in vitro evidence to support this assumption. The second potential explanation is that the infiltrating TAM density may not have been sufficient to exert an overwhelming killing effect that could suppress cancer cell growth and metastasis. Ma et al. reported a mean TAM density of 168.3 mm^-2^ (range, 0–554.9 mm^-2^) within lung cancer tissue [77], which is a greater density than the TAM density (mean, 40.3 mm^-2^; range, 3.3 - 159.7 mm^-2^) that we detected in ovarian cancer tissue (Additional file 1: Table S1). Therefore, the low intratumoral TAM density somewhat weakens the TAM density theory in the ovarian cancer cases studied. The third explanation is that the M2 TAMs detected in ovarian cancer tissue might retain their original nutritional effect on cancer cells, which could compete against the anticancer effect of their M1 counterparts. As such, no prognostic value of the increased total TAM density was observed in the study.

Both the M1- and M2-dominant theories were formulated after the roles of the classically or alternatively activated macrophages in killing or maintaining in vitro cultured cancer cells were demonstrated [78,79]. Many clinical studies have correlated improved prognosis with increased M1 TAM and decreased M2 TAM densities in various human malignancies [40,47,71,80]. Similarly, we demonstrated an improved long-term survival effect of M1 TAMs in ovarian cancer patients, as assessed using the multivariate Cox regression model (Additional file 1: Tables S4 and S5). However, for M1 TAMs, only the increased cell density in cancer islets reached significance for improved prognosis, while the increase in intra-stromal M1 TAM density did not affect patient outcome. Previously, Ohri et al. argued that mature macrophages with anticancer activity must interact intimately with target cancer cells to exert their tumoricidal properties, which could logically underlie the clinical significance of infiltrating M1 TAMs [30]. From this perspective, our findings provide further evidence supporting Ohri’s theory. Nevertheless, our results were obtained after normalizing several confounding factors such as patient age, cancer stage, histotype, and grade, and we also excluded the prognostic effects from their antagonizing counterparts such as M2 TAMs. Moreover, no single M1- or M2-related parameter provided prognostic significance under more complicated conditions such as Kaplan-Meier survival analysis. Thus, in contrast to patients with pancreatic, esophageal, and breast cancer, increased M1 TAM density alone cannot achieve a predominant prognostic value in ovarian cancer patients [16,40,41]. These pathological findings indicate that the long-term effects of M1 or M2 TAMs function in a more complicated manner in ovarian cancer, which does not simply comply with the known M1- and M2-dominant rules.

Among all the investigated TAM-related parameters, we only demonstrated that the increased overall and intra-islet M1/M2 TAM ratios possessed the prognostic significance in both the Kaplan-Meier survival and multivariate Cox regression analyses. These findings are tenable because the biological effects of M1 and M2 TAMs antagonize each other, and neither TAM type could create a predominant effect in all the examined cancer tissues. Furthermore, we noted that the intra-stromal M1/M2 TAM ratio did not possess any statistical prognostic significance. Considering Ohri’s theory and the fact that intra-stromal M1 TAM density was not an independent factor influencing the 5-year cancer survival (Additional file 1: Table S3), this result is rational, as it demonstrates the necessity of the direct interaction between TAMs and cancer cells to create a tumoricidal effect. In addition, we noted that the prognostic value of the intra-islet M1/M2 TAM ratio varied between histotypes, suggesting that the tumoricidal effects of M1 TAMs or nutritional effects of M2 TAMs were altered in various ovarian cancer histotypes. Endometrioid ovarian cancer is less malignant than most other ovarian cancer histotypes including serous, clear cell, and undifferentiated cancers [2,21-24]. Similarly, the infiltration density of TAMs was much lower in endometrioid ovarian cancer tissue compared with other ovarian cancer histotypes. Therefore, the M1/M2 ratio of the intratumoral TAMs may play a less prominent role in endometrioid ovarian cancer progression than the M1/M2 ratio observed in other ovarian cancer histotypes. Regarding the serous ovarian cancer histotype, progression occurs more quickly and often induces vigorous TAM infiltration into the cancer tissue bed. These serous cancer cells could be more sensitive to the killing or nutritional effects of M1 or M2 TAMs, respectively, than other cancer cell histotypes. Therefore, the M1/M2 TAM ratio exerted the greatest influence and led to the highest HR in our study. Our results are consistent with previous reports demonstrating the prognostic value of the M1/M2 TAM ratio in several other malignancies including glioma, melanoma, prostate cancer, and liver metastases of colorectal cancer [64,81-83]. These cancers and ovarian cancer should possess similar TAM response properties.

The histotype-specific roles of TAMs in the progression of ovarian cancer have been carefully evaluated in our study. Although our data demonstrated that the overall and intra-islet M1/M2 TAM ratios are independent prognostic factors for patient survival, we could not establish an effective relationship between the histotype-specific HRs of these two ratios and the 5-year survival rates of patients (Table 5 and Figure 4). These data suggest that the biological effects of TAMs, which are differentially exerted on each cancer histotype, were not robust enough to independently alter the overall clinical behavior of an ovarian cancer histotype. Altered TAM distribution patterns such as the TAM densities (except for the overall, intra-islet and intra-stromal M2 TAM densities) and the islet/stroma ratio of total TAMs (Table 2) that were tightly associated with cancer histotypes did not effectively influence the outcome of an individual patient (Figure 3) and they were not correlated with or the overall 5-year survival rates of different histotypes of ovarian cancer (Table 2 and Additional file 1: Tables S4 and S5) histotype. Moreover, multivariate Cox regression analysis indicated that the major factors that determine cancer patient fate still resided in several main clinical-pathological characteristics (Table 3 and Additional file 1: Tables S1-S14). Compared with the HRs of cancer stage, histotype, grade, and the size of the residual site, none of the parameters related to density, differentiation, or microlocalization of TAMs (including the overall and intra-islet M1 TAM densities) reached a higher HR value, indicating that all of the investigated TAM parameters have a minor influence on disease progression. Based on these TAM parameter analyses, we conclude that TAM distribution patterns can only be utilized to predict the outcome of an individual patient, but they are not applicable for explaining the different outcomes observed between cancer histotypes. These different outcomes should still be attributed to the characteristic proliferation, migration, invasion, and anti-apoptosis ability possessed by ovarian cancer cells of a particular histotype.

## Conclusions

In conclusion, the present study demonstrates that the overall and intra-islet M1/M2 ratios are effective TAM parameters to predict the outcomes of ovarian cancer patients, especially for those with a serous histotype. Although the total and M1 TAM densities and the islet/stroma ratio of total TAMs exhibited ovarian cancer histotype-specific characteristics, patient prognosis is determined by the dominant TAM phenotype in the cancer tissue, which showed no special distribution patterns among the cancer histotypes. Moreover, for M1 TAMs to exert their anticancer effects on ovarian cancer cells, it would be better if they reside in the islet region and maintain contact with the cancer cells. Our observations implicate the necessity for personalized immunomodulatory therapy in ovarian cancer patients with different TAM distribution statuses, which may promote M1 polarization of TAMs and accelerate their intra-islet infiltration; therefore, the long-term survival of the patients could be improved.

## Abbreviations

TAM: Tumor-associated macrophages; LCM: Laser capture microdissection.

## Competing interests

The authors declare that they have no competing interests.

## Authors’ contributions

YFH, AMZ, and WD designed the study. MYZ performed the immunohistochemistry and immunofluorescence work. YFH and XJS independently reviewed the immunostained cancer tissue sections. WJW and QL performed the LCM and flow cytometry. YFH wrote the manuscript. All authors read and approved the final manuscript.

## Supplementary Material

Additional file 1: Tables S1-S14 Describe the multivariate Cox regression analytic results for the following TAM-related parameters (for the overall M1/M2 TAM ratio, see Table 4): total TAM density **(Table S1)**; intra-islet TAM density **(Table S2)**; intra-stromal TAM density **(Table S3)**; overall M1 TAM density **(Table S4)**; intra-islet M1 TAM density **(Table S5)**; intra-stromal M1 TAM density **(Table S6)**; overall M2 TAM density **(Table S7)**; intra-islet M2 TAM density **(Table S8)**; intra-stromal M2 TAM density **(Table S9)**; islet/stroma ratio of total TAMs **(Table S10)**; islet/stroma ratio of M1 TAMs **(Table S11)**; islet/stroma ratio of M2 TAMs **(Table S12)**; intra-islet M1/M2 TAM ratio **(Table S13)**; and intra-stromal M1/M2 TAM ratio **(Table S14)**. Click here for file
