# Supplementary material for: A high M1/M2 ratio of tumor-associated macrophages is associated with extended survival in ovarian cancer patients
Source: J Ovarian Res. 2014 Feb 8;7:19. doi: 10.1186/1757-2215-7-19 (PMC3939626; doi:10.1186/1757-2215-7-19)
Supplement: Additional file 1: Tables S1-S14 — Describe the multivariate Cox regression analytic results for the following TAM-related parameters (for the overall M1/M2 TAM ratio, see Table 4): total TAM density (Table S1); intra-islet TAM density (Table S2); intra-stromal TAM density (Table S3); overall M1 TAM density (Table S4); intra-islet M1 TAM density (Table S5); intra-stromal M1 TAM density (Table S6); overall M2 TAM density (Table S7); intra-islet M2 TAM density (Table S8); intra-stromal M2 TAM density (Table S9); islet/stroma ratio of total TAMs (Table S10); islet/stroma ratio of M1 TAMs (Table S11); islet/stroma ratio of M2 TAMs (Table S12); intra-islet M1/M2 TAM ratio (Table S13); and intra-stromal M1/M2 TAM ratio (Table S14). [file 1757-2215-7-19-S1.pdf]

Supplementary Tables

Table S1. Multivariate Cox regression analysis of clinical, pathological and TAM-related prognostic factors for ovarian cancer. (Note: the total TAM density is involved)

Parameters	Hazard ratio	95% confidence interval	p value
Age			0.437
<40	1 (reference)	-	
40-49	0.635	(0.119, 3.393)	
50-59	0.785	(0.164, 3.758)	
60-69	0.580	(0.125, 2.691)	
≥70	0.177	(0.022, 1.408)	
Gravidity			0.870
0-1	1 (reference)	-	
2-3	1.291	(0.566, 2.946)	
4-5	0.962	(0.401, 2.306)	
≥5	1.065	(0.323, 3.507)	
Parity			0.491
0-1	1 (reference)	-	
2-3	0.791	(0.406, 1.540)	
Menopause			0.875
Yes	1 (reference)	-	
No	0.933	(0.389, 2.236)	
Ascites			0.113
Yes	1 (reference)	-	
No	0.395	(0.125, 1.248)	
Peritoneal metastasis			<0.001[#]
Yes	1 (reference)	-	
No	8.547	(2.978, 24.534)	
Lymphatic metastasis			0.779
Yes	1 (reference)	-	
No	0.864	(0.312, 2.394)	
Stage			<0.001[#]
I	1 (reference)	-	
II	2.8	(0.550, 14.243)	
III	8.8	(2.433, 31.829)	
IV	100.0	(15.784, 633.525)	
Histotype			0.004[#]

Serous	1 (reference)	-	
Mucinous	0.101	(0.014, 0.746)	
Endometrioid	0.368	(0.079, 1.707)	
Clear cell	0.598	(0.115, 3.103)	
Undifferentiated	2.646	(0.586, 11.945)	
Grade			0.001#
G1	1 (reference)	-	
G2	6.176	(2.893, 13.186)	
G3	13.514	(3.474, 52.563)	
Size of residual site			0.049#
< 2 cm	1 (reference)	-	
≥ 2 cm	2.198	(1.004, 4.814)	
Total TAM density			0.079
<40.313	1 (reference)	-	
≥40.313	0.987	(0.972, 1.002)	

Statistical significance

Table S2. Multivariate Cox regression analysis of clinical, pathological and TAM-related prognostic factors for ovarian cancer. (Note: the intra-islet TAM density is involved)

Parameters	Hazard ratio	95% confidence interval	p value
Age			0.432
<40	1 (reference)	-	
40-49	0.623	(0.177, 2.201)	
50-59	0.753	(0.188, 3.021)	
60-69	0.594	(0.125, 2.830)	
≥70	0.171	(0.022, 1.342)	
Gravidity			0.863
0-1	1 (reference)	-	
2-3	1.298	(0.457, 3.686)	
4-5	0.954	(0.303, 3.004)	
≥5	1.115	(0.336, 3.703)	
Parity			0.439
0-1	1 (reference)	-	
2-3	0.769	(0.395, 1.497)	
Menopause			0.991
Yes	1 (reference)	-	
No	1.005	(0.421, 2.400)	
Ascites			0.126
Yes	1 (reference)	-	
No	0.399	(0.123,1.294)	
Peritoneal metastasis			＜0.001#
Yes	1 (reference)	-	
No	8.268	(2.889, 23.667)	
Lymphatic metastasis			0.626
Yes	1 (reference)	-	
No	0.772	(0.272, 2.190)	
Stage			＜0.001#
I	1 (reference)	-	
II	2.6	(0.970, 7.203)	
III	8.7	(2.390, 31.863)	
IV	96.3	(15.294, 606.836)	
Histotype			0.003#
Serous	1 (reference)	-	

Mucinous	0.083	(0.018, 0.377)	
Endometrioid	0.401	(0.158, 1.019)	
Clear cell	0.631	(0.227, 1.750)	
Undifferentiated	2.872	(0.644, 12.812)	
Grade			0.001#
G1	1 (reference)	-	
G2	5.801	(1.814, 18.546)	
G3	12.112	(3.241, 45.262)	
Size of residual site			0.045#
< 2 cm	1 (reference)	-	
≥ 2 cm	2.214	(1.018, 4.814)	
Intra-islet TAM density			0.102
<18.955	1 (reference)	-	
≥18.955	0.975	(0.945, 1.005)	

Statistical significance

Table S3. Multivariate Cox regression analysis of clinical, pathological and TAM-related prognostic factors for ovarian cancer. (Note: the intra-stromal TAM density is involved)

Parameters	Hazard ratio	95% confidence interval	p value
Age			0.365
<40	1 (reference)	-	
40-49	0.666	(0.124, 3.579)	
50-59	0.866	(0.182, 4.130)	
60-69	0.582	(0.123, 2.743)	
≥70	0.179	(0.023, 1.424)	
Gravidity			0.853
0-1	1 (reference)	-	
2-3	1.244	(0.546, 2.834)	
4-5	0.913	(0.382, 2.184)	
≥5	0.979	(0.299, 3.211)	
Parity			0.536
0-1	1（reference）	-	
2-3	0.810	(0.415, 1.580)	
Menopause			0.802
Yes	1 (reference)	-	
No	0.894	(0.371, 2.155)	
Ascites			0.121
Yes	1 (reference)	-	
No	0.414	(0.136, 1.263)	
Peritoneal metastasis			＜0.001[#]
Yes	1 (reference)	-	
No	8.696	(3.041, 24.864)	
Lymphatic metastasis			0.952
Yes	1 (reference)	-	
No	0.970	(0.356, 2.641)	
Stage			＜0.001[#]
I	1 (reference)	-	
II	3.2	(0.666, 15.381)	
III	8.7	(2.443, 30.982)	
IV	100.0	(16.413, 609.287)	
Histotype			0.006[#]
Serous	1 (reference)	-	

Mucinous	0.118	(0.158, 0.883)	
Endometrioid	0.368	(0.079, 1.711)	
Clear cell	0.624	(0.121, 3.225)	
Undifferentiated	2.688	(0.607, 11.899)	
Grade			0.001[#]
G1	1 (reference)	-	
G2	6.224	(2.955, 13.108)	
G3	13.158	(3.430, 50.481)	
Size of residual site			0.049[#]
< 2 cm	1 (reference)	-	
≥ 2 cm	2.193	(1.003, 4.794)	
Intra-stromal TAM density			0.097
<21.357	1 (reference)	-	
≥21.357	0.979	(0.955, 1.004)	

Statistical significance.

Table S4. Multivariate Cox regression analysis of clinical, pathological and TAM-related prognostic factors for ovarian cancer. (Note: the overall M1 TAM density is involved)

Parameters	Hazard ratio	95% confidence interval	p value
Age			0.585
<40	1 (reference)	-	
40-49	0.575	(0.108, 3.048)	
50-59	0.790	(0.170, 3.665)	
60-69	0.684	(0.155, 3.022)	
≥70	0.254	(0.033, 1.977)	
Gravidity			0.957
0-1	1 (reference)	-	
2-3	1.257	(0.548, 2.886)	
4-5	1.129	(0.461, 2.765)	
≥5	1.044	(0.330, 3.305)	
Parity			0.600
0-1	1 (reference)	-	
2-3	0.837	(0.431, 1.627)	
Menopause			0.749
Yes	1 (reference)	-	
No	0.867	(0.360, 2.086)	
Ascites			0.054
Yes	1 (reference)	-	
No	0.316	(0.098, 1.016)	
Peritoneal metastasis			<0.001[#]
Yes	1 (reference)	-	
No	9.901	(3.389, 28.926)	
Lymphatic metastasis			0.687
Yes	1 (reference)	-	
No	0.805	(0.278, 2.329)	
Stage			<0.001[#]
I	1 (reference)	-	
II	3.222	(0.657, 15.793)	
III	12.333	(3.484, 43.662)	
IV	111.111	(17.639, 699.923)	
Histotype			0.004[#]
Serous	1 (reference)	-	

Mucinous	0.119	(0.016, 0.879)	
Endometrioid	0.311	(0.066, 1.474)	
Clear cell	0.461	(0.087, 2.444)	
Undifferentiated	2.532	(0.548, 11.702)	
Grade			<0.001#
G1	1 (reference)	-	
G2	7.082	(3.158, 15.880)	
G3	20.408	(4.702, 88.585)	
Size of residual site			0.044#
< 2 cm	1 (reference)	-	
≥ 2 cm	2.273	(1.022, 5.057)	
Overall M1 TAM density			0.007#
<22.321	1 (reference)	-	
≥22.321	0.960	(0.933, 0.989)	

Statistical significance

Table S5. Multivariate Cox regression analysis of clinical, pathological and TAM-related prognostic factors for ovarian cancer. (Note: the intra-islet M1 TAM density is involved)

Parameters	Hazard ratio	95% confidence interval	p value
Age			0.402
<40	1 (reference)	-	
40-49	0.393	(0.077, 1.999)	
50-59	0.482	(0.110, 2.113)	
60-69	0.573	(0.137, 2.392)	
≥70	0.181	(0.024, 1.379)	
Gravidity			0.905
0-1	1 (reference)	-	
2-3	1.455	(0.623, 3.295)	
4-5	1.345	(0.539, 3.359)	
≥5	1.263	(0.401, 3.975)	
Parity			0.417
0-1	1 (reference)	-	
2-3	0.762	(0.408, 1.472)	
Menopause			0.941
Yes	1 (reference)	-	
No	1.034	(0.424, 2.522)	
Ascites			0.022#
Yes	1 (reference)	-	
No	0.230	(0.066, 0.805)	
Peritoneal metastasis			<0.001#
Yes	1 (reference)	-	
No	9.709	(3.227, 29.212)	
Lymphatic metastasis			0.245
Yes	1 (reference)	-	
No	0.524	(0.176, 1.561)	
Stage			<0.001#
I	1 (reference)	-	
II	2.833	(0.513, 15.650)	
III	16.833	(4.527, 62.586)	
IV	166.667	(23.066, 1204.281)	
Histotype			0.002#
Serous	1 (reference)	-	

Mucinous	0.095	(0.013, 0.708)	
Endometrioid	0.343	(0.073, 1.601)	
Clear cell	0.450	(0.085, 2.395)	
Undifferentiated	2.725	(0.591, 12.569)	
Grade			<0.001#
G1	1 (reference)	-	
G2	6.660	(3.083, 14.388)	
G3	18.868	(4.646, 76.621)	
Size of residual site			0.092
< 2 cm	1 (reference)	-	
≥ 2 cm	1.976	(0.893, 4.370)	
Intra-islet M1 TAM density			0.001#
<11.330	1 (reference)	-	
≥11.330	0.914	(0.866, 0.965)	

Statistical significance.

Table S6. Multivariate Cox regression analysis of clinical, pathological and TAM-related prognostic factors for ovarian cancer. (Note: the intra-stromal M1 TAM density is involved)

Parameters *	Hazard ratio†	95% confidence interval	p value
Age			0.273
<40	1 (reference)	-	
40-49	0.782	(0.146, 4.186)	
50-59	1.152	(0.247, 5.366)	
60-69	0.736	(0.159, 3.415)	
≥70	0.217	(0.027, 1.733)	
Gravidity			0.844
0-1	1 (reference)	-	
2-3	1.117	(0.488, 2.559)	
4-5	0.813	(0.340, 1.945)	
≥5	0.864	(0.266, 2.812)	
Parity			0.607
0-1	1 (reference)	-	
2-3	0.839	(0.429, 1.640)	
Menopause			0.838
Yes	1 (reference)	-	
No	0.912	(0.380, 2.190)	
Ascites			0.177
Yes	1 (reference)	-	
No	0.475	(0.161, 1.399)	
Peritoneal metastasis			<0.001#
Yes	1 (reference)	-	
No	9.259	(3.277, 26.164)	
Lymphatic metastasis			0.902
Yes	1 (reference)	-	
No	1.066	(0.390, 2.914)	
Stage			<0.001#
I	1 (reference)	-	
II	3.063	(0.730, 12.860)	
III	6.688	(1.969, 22.722)	
IV	62.500	(11.999, 325.540)	
Histotype			0.006#
Serous	1 (reference)	-	

Mucinous	0.118	(0.016, 0.858)	
Endometrioid	0.415	(0.088, 1.964)	
Clear cell	0.696	(0.134, 3.604)	
Undifferentiated	3.268	(0.765, 13.965)	
Grade			0.001#
G1	1 (reference)	-	
G2	6.085	(2.918, 12.690)	
G3	12.195	(3.179, 46.786)	
Size of residual site			0.029#
< 2 cm	1 (reference)	-	
≥ 2 cm	2.358	(1.089, 5.104)	
Intra-stromal M1 TAM density			0.184
<10.991	1 (reference)	-	
≥10.991	0.973	(0.934, 1.013)	

Statistical significance

Table S7. Multivariate Cox regression analysis of clinical, pathological and TAM-related prognostic factors for ovarian cancer. (Note: the overall M2 TAM density is involved)

Parameters	Hazard ratio	95% confidence interval	p value
Age			0.230
<40	1 (reference)	-	
40-49	0.709	(0.136, 3.700)	
50-59	0.942	(0.201, 4.414)	
60-69	0.600	(0.130, 2.778)	
≥70	0.153	(0.020, 1.182)	
Gravidity			0.715
0-1	1 (reference)	-	
2-3	1.177	(0.513, 2.702)	
4-5	0.768	(0.325, 1.816)	
≥5	0.923	(0.271, 3.148)	
Parity			0.491
0-1	1 (reference)	-	
2-3	0.791	(0.405, 1.543)	
Menopause			0.984
Yes	1 (reference)	-	
No	1.009	(0.423, 2.409)	
Ascites			0.202
Yes	1 (reference)	-	
No	0.485	(0.159, 1.476)	
Peritoneal metastasis			<0.001[#]
Yes	1 (reference)	-	
No	8.130	(2.911, 22.705)	
Lymphatic metastasis			0.876
Yes	1 (reference)	-	
No	0.925	(0.349, 2.450)	
Stage			<0.001[#]
I	1 (reference)	-	
II	2.786	(0.567, 13.683)	
III	6.286	(1.762, 22.429)	
IV	71.429	(11.932, 427.598)	
Histotype			0.004[#]
Serous	1 (reference)	-	

Mucinous	0.089	(0.012, 0.641)	
Endometrioid	0.479	(0.104, 2.201)	
Clear cell	0.809	(0.160, 4.092)	
Undifferentiated	3.300	(0.765, 14.240)	
Grade			0.002#
G1	1 (reference)	-	
G2	5.382	(2.721, 10.646)	
G3	9.091	(2.676, 30.887)	
Size of residual site			0.035#
< 2 cm	1 (reference)	-	
≥ 2 cm	2.262	(1.058, 4.839)	
Overall M2 TAM density			0.653
<17.991	1 (reference)	-	
≥17.991	0.994	(0.971, 1.019)	

Statistical significance

Table S8. Multivariate Cox regression analysis of clinical, pathological and TAM-related prognostic factors for ovarian cancer. (Note: the intra-islet M2 TAM density is involved)

Parameters	Hazard ratio	95% confidence interval	p value
Age			0.117
<40	1 (reference)	-	
40-49	0.674	(0.131, 3.456)	
50-59	1.097	(0.239, 5.030)	
60-69	0.634	(0.141, 2.856)	
≥70	0.146	(0.020, 1.072)	
Gravidity			0.622
0-1	1 (reference)	-	
2-3	1.062	(0.457, 2.467)	
4-5	0.675	(0.283, 1.608)	
≥5	0.755	(0.221, 2.575)	
Parity			0.508
0-1	1 (reference)	-	
2-3	0.791	(0.408, 1.534)	
Menopause			0.922
Yes	1 (reference)	-	
No	1.009	(0.421, 2.418)	
Ascites			0.201
Yes	1 (reference)	-	
No	0.485	(0.167, 1.409)	
Peritoneal metastasis			＜0.001#
Yes	1 (reference)	-	
No	8.130	(2.946, 22.440)	
Lymphatic metastasis			0.885
Yes	1 (reference)	-	
No	0.925	(0.363, 2.356)	
Stage			＜0.001#
I	1 (reference)	-	
II	2.786	(0.656, 11.835)	
III	6.286	(1.891, 20.901)	
IV	71.429	(14.067, 362.688)	
Histotype			0.004#
Serous	1 (reference)	-	

Mucinous	0.089	(0.013, 0.618)	
Endometrioid	0.479	(0.104, 2.205)	
Clear cell	0.809	(0.167, 4.028)	
Undifferentiated	3.300	(0.789, 13.801)	
Grade			0.006#
G1	1 (reference)	-	
G2	5.382	(2.737, 10.583)	
G3	9.091	(2.772, 29.816)	
Size of residual site			0.026#
< 2 cm	1 (reference)	-	
≥ 2 cm	2.262	(1.080, 4.736)	
Intra-islet M2 TAM density			0.288
<7.625	1 (reference)	-	
≥7.625	1.029	(0.976, 1.083)	

Statistical significance.

Table S9. Multivariate Cox regression analysis of clinical, pathological and TAM-related prognostic factors for ovarian cancer. (Note: the intra-stromal M2 TAM density is involved)

Parameters	Hazard ratio	95% confidence interval	p value
Age			0.284
<40	1 (reference)	-	
40-49	0.612	(0.115, 3.270)	
50-59	0.743	(0.157, 3.516)	
60-69	0.501	(0.107, 2.347)	
≥70	0.136	(0.017, 1.101)	
Gravidity			0.758
0-1	1 (reference)	-	
2-3	1.290	(0.567, 2.933)	
4-5	0.863	(0.362, 2.056)	
≥5	1.013	(0.303, 3.388)	
Parity			0.446
0-1	1 (reference)	-	
2-3	0.770	(0.393, 1.538)	
Menopause			0.933
Yes	1(reference)	-	
No	0.963	(0.401, 2.313)	
Ascites			0.130
Yes	1(reference)	-	
No	0.415	(0.133, 1.293)	
Peritoneal metastasis			<0.001#
Yes	1(reference)	-	
No	8.065	(2.826, 23.015)	
Lymphatic metastasis			0.790
Yes	1(reference)	-	
No	0.875	(0.326, 2.345)	
Stage			<0.001#
I	1(reference)	-	
II	3.111	(0.595, 16.268)	
III	8.444	(2.271, 31.395)	
IV	111.111	(16.861, 732.197)	
Histotype			0.006#
Serous	1(reference)	-	

Mucinous	0.102	(0.014, 0.756)	
Endometrioid	0.418	(0.091, 1.913)	
Clear cell	0.709	(0.139, 3.628)	
Undifferentiated	2.770	(0.639, 12.000)	
Grade			0.001#
G1	1 (reference)	-	
G2	5.629	(2.829, 11.200)	
G3	10.309	(3.011, 35.301)	
Size of residual site			0.061
< 2 cm	1 (reference)	-	
≥ 2 cm	2.105	(0.967, 4.583)	
Intra-stromal M2 TAM density			0.175
<10.366	1 (reference)	-	
≥10.366	0.972	(0.934,1.013)	

Statistical significance

Table S10. Multivariate Cox regression analysis of clinical, pathological and TAM-related prognostic factors for ovarian cancer. (Note: the islet/stroma ratio of total TAMs is involved)

Parameters	Hazard ratio	95% confidence interval	p value
Age			0.151
<40	1(reference)	-	
40-49	0.757	(0.144,3.990)	
50-59	1.133	(0.243,5.278)	
60-69	0.654	(0.141,3.029)	
≥70	0.163	(0.021,1.256)	
Gravidity			0.645
0-1	1(reference)	-	
2-3	1.111	(0.484,2.551)	
4-5	0.704	(0.298,1.664)	
≥5	0.810	(0.241,2.720)	
Parity			0.560
0-1	1（reference）	-	
2-3	0.821	(0.423,1.592)	
Menopause			0.901
Yes	1(reference)	-	
No	0.945	(0.388,2.301)	
Ascites			0.190
Yes	1(reference)	-	
No	0.483	(0.163,1.431)	
Peritoneal metastasis			＜0.001#
Yes	1(reference)	-	
No	8.333	(3.007,23.091)	
Lymphatic metastasis			0.988
Yes	1(reference)	-	
No	0.993	(0.376,2.620)	
Stage			＜0.001#
I	1(reference)	-	
II	3.286	(0.786,13.742)	
III	6.643	(1.940,22.747)	
IV	71.429	(13.527,377.188)	
Histotype			0.012#
Serous	1(reference)	-	

Mucinous	0.109	(0.014,0.867)	
Endometrioid	0.492	(0.106,2.287)	
Clear cell	0.863	(0.165,4,513)	
Undifferentiated	3.115	(0.732,13.259)	
Grade			0.001#
G1	1(reference)	-	
G2	5.205	(2.726,9.939)	
G3	8.547	(2.647,27.596)	
Size of residual site			0.026#
< 2 cm	1(reference)	-	
≥ 2 cm	2.358	(1.109,5.015)	
Islet/stromal ratio of total TAMs			0.415
<1.049	1(reference)	-	
≥1.049	1.342	(0.662,2.719)	

Statistical significance.

Table S11. Multivariate Cox regression analysis of clinical, pathological and TAM-related prognostic factors for ovarian cancer. (Note: the islet/stroma ratio of M1 TAMs is involved)

Parameters *	Hazard ratio†	95% confidence interval	p value
Age			0.171
<40	1 (reference)	-	
40-49	0.764	(0.147, 3.972)	
50-59	1.004	(0.222, 4.550)	
60-69	0.593	(0.130, 2.714)	
≥70	0.153	(0.020, 1.154)	
Gravidity			0.677
0-1	1 (reference)	-	
2-3	1.174	(0.503, 2.738)	
4-5	0.752	(0.312, 1.813)	
≥5	0.946	(0.275, 3.258)	
Parity			0.409
0-1	1 (reference)	-	
2-3	0.751	(0.380, 1.483)	
Menopause			0.857
Yes	1 (reference)	-	
No	1.086	(0.445, 2.649)	
Ascites			0.221
Yes	1 (reference)	-	
No	0.507	(0.171, 1.502)	
Peritoneal metastasis			<0.001#
Yes	1 (reference)	-	
No	8.264	(2.947, 23.125)	
Lymphatic metastasis			0.977
Yes	1 (reference)	-	
No	0.986	(0.368, 2.643)	
Stage			<0.001#
I	1 (reference)	-	
II	2.875	(0.675, 12.237)	
III	5.688	(1.661, 19.477)	
IV	62.500	(12.023, 324.903)	
Histotype			0.007#
Serous	1 (reference)	-	
Mucinous	0.095	(0.013, 0.682)	

Endometrioid	0.556	(0.115, 2.683)	
Clear cell	0.867	(0.169, 4.437)	
Undifferentiated	3.175	(0.756, 13.330)	
Grade			0.001#
G1	1 (reference)	-	
G2	4.957	(2.566, 9.577)	
G3	8.547	(2.679, 27.273)	
Size of residual site			0.028#
< 2 cm	1 (reference)	-	
≥ 2 cm	2.336	(1.094, 4.988)	
Islet/stromal ratio of M1 TAMs			0.630
<1.453	1 (reference)	-	
≥1.453	1.053	(0.854, 1.299)	

Statistical significance.

Table S12. Multivariate Cox regression analysis of clinical, pathological and TAM-related prognostic factors for ovarian cancer. (Note: the islet/stroma ratio of M2 TAMs is involved)

Parameters	Hazard ratio	95% confidence interval	p value
Age			0.142
<40	1 (reference)	-	
40-49	0.790	(0.153, 4.075)	
50-59	1.139	(0.255, 5.092)	
60-69	0.617	(0.138, 2.758)	
≥70	0.174	(0.023, 1.336)	
Gravidity			0.699
0-1	1 (reference)	-	
2-3	1.227	(0.541, 2.784)	
4-5	0.813	(0.339, 1.949)	
≥5	0.883	(0.271, 2.879)	
Parity			0.543
0-1	1 (reference)	-	
2-3	0.813	(0.418, 1.583)	
Menopause			0.843
Yes	1 (reference)	-	
No	0.915	(0.122, 2.197)	
Ascites			0.108
Yes	1 (reference)	-	
No	0.418	(0.144, 1.209)	
Peritoneal metastasis			<0.001[#]
Yes	1 (reference)	-	
No	7.463	(2.657, 20.965)	
Lymphatic metastasis			0.667
Yes	1 (reference)	-	
No	0.812	(0.315, 2.093)	
Stage			<0.001[#]
I	1 (reference)	-	
II	3.8	(0.898, 16.080)	
III	9.7	(2.878, 32.698)	
IV	100.0	(17.441, 573.369)	
Histotype			0.044[#]
Serous	1 (reference)	-	

Mucinous	0.144	(0.018, 1.125)	
Endometrioid	0.487	(0.103, 2.313)	
Clear cell	0.819	(0.154, 4.359)	
Undifferentiated	2.353	(0.545, 10.154)	
Grade			0.003#
G1	1 (reference)	-	
G2	4.809	(2.504, 9.237)	
G3	7.634	(2.388, 24.408)	
Size of residual site			0.068
< 2 cm	1 (reference)	-	
≥ 2 cm	2.062	(0.947, 4.490)	
Islet/stromal ratio of M2 TAMs			0.019#
<0.936	1 (reference)	-	
≥0.936	2.031	(1.125, 3.666)	

Statistical significance

Table S13. Multivariate Cox regression analysis of clinical, pathological and TAM-related prognostic factors for ovarian cancer. (Note: the intra-islet M1/M2 TAM ratio is involved)

Parameters	Hazard ratio	95% confidence interval	p value
Age			0.225
<40	1 (reference)	-	
40-49	0.709	(0.145, 3.462)	
50-59	1.112	(0.263, 4.696)	
60-69	0.817	(0.195, 3.423)	
≥70	0.212	(0.029, 1.529)	
Gravidity			0.770
0-1	1 (reference)	-	
2-3	1.127	(0.484, 2.623)	
4-5	0.909	(0.355, 2.324)	
≥5	0.723	(0.225, 2.321)	
Parity			0.836
0-1	1 (reference)	-	
2-3	0.931	(0.474, 1.827)	
Menopause			0.932
Yes	1 (reference)	-	
No	0.962	(0.402, 2.301)	
Ascites			0.113
Yes	1 (reference)	-	
No	0.409	(0.135, 1.235)	
Peritoneal metastasis			<0.001#
Yes	1 (reference)	-	
No	8.929	(3.185, 25.035)	
Lymphatic metastasis			0.557
Yes	1 (reference)	-	
No	0.746	(0.282, 1.976)	
Stage			<0.001#
I	1 (reference)	-	
II	3.4	(0.813, 14.219)	
III	7.8	(2.305, 26.397)	
IV	62.5	(11.976, 326.179)	
Histotype			0.003#
Serous	1 (reference)	-	

Mucinous	0.117	(0.017, 0.810)	
Endometrioid	0.519	(0.111, 2.432)	
Clear cell	0.864	(0.164, 4.554)	
Undifferentiated	4.673	(1.113, 19.619)	
Grade			0.004#
G1	1 (reference)	-	
G2	4.399	(2.272, 8.400)	
G3	7.246	(2.249, 23.349)	
Size of residual site			0.048#
< 2 cm	1 (reference)	-	
≥ 2 cm	2.119	(1.006, 4.463)	
Intra-islet M1/M2 TAM ratio			0.038#
<1.752	1 (reference)	-	
≥1.752	0.644	(0.425, 0.976)	

Statistical significance

Table S14. Multivariate Cox regression analysis of clinical, pathological and TAM-related prognostic factors for ovarian cancer. (Note: the intra-stromal M1/M2 TAM ratio is involved)

Parameters	Hazard ratio	95% confidence interval	p value
Age			0.179
<40	1 (reference)	-	
40-49	0.802	(0.154, 4.177)	
50-59	1.191	(0.262, 5.419)	
60-69	0.750	(0.165, 3.412)	
≥70	0.187	(0.024, 1.470)	
Gravidity			0.730
0-1	1 (reference)	-	
2-3	1.102	(0.473, 2.570)	
4-5	0.751	(0.302, 1.865)	
≥5	0.794	(0.235, 2.687)	
Parity			0.658
0-1	1 (reference)	-	
2-3	0.857	(0.432, 1.698)	
Menopause			0.992
Yes	1 (reference)	-	
No	0.995	(0.417, 2.376)	
Ascites			0.214
Yes	1 (reference)	-	
No	0.501	(0.168, 1.490)	
Peritoneal metastasis			<0.001[#]
Yes	1 (reference)	-	
No	8.850	(3.144, 24.911)	
Lymphatic metastasis			0.961
Yes	1 (reference)	-	
No	0.976	(0.368, 2.585)	
Stage			<0.001[#]
I	1 (reference)	-	
II	2.9	(0.635, 13.246)	
III	5.5	(1.563, 19.319)	
IV	52.6	(9.787, 282.703)	
Histotype			0.004[#]
Serous	1 (reference)	-	

Mucinous	0.096	(0.014, 0.680)	
Endometrioid	0.526	(0.112, 2.469)	
Clear cell	0.845	(0.163, 4.376)	
Undifferentiated	3.984	(0.942, 16.858)	
Grade			0.001#
G1	1 (reference)	-	
G2	5.228	(2.722, 10.041)	
G3	8.772	(2.696, 28.558)	
Size of residual site			0.019#
< 2 cm	1 (reference)	-	
≥ 2 cm	2.538	(1.166, 5.526)	
Intra-stromal M1/M2 TAM ratio			0.424
<1.261	1 (reference)	-	
≥1.261	0.826	(0.516, 1.320)	

Statistical significance.
